# Morphometric assessment of blastocysts: relationship with the ongoing pregnancy rate

**DOI:** 10.1016/j.xfre.2022.11.001

**Published:** 2022-11-11

**Authors:** Hiroki Utsuno, Tomoko Ishimaru, Miho Matsumoto, Chiharu Sasamori, Hikaru Takahashi, Hiroko Kimura, Shintaro Kamijo, Mitsutoshi Yamada, Mamoru Tanaka, Toshio Hamatani

**Affiliations:** a Clinical Laboratory, Keio University Hospital, Tokyo, Japan; b Department of Obstetrics and Gynecology, Keio University School of Medicine, Tokyo, Japan

**Keywords:** Blastocyst morphology, morphometry, ongoing pregnancy rate, inner cell mass, trophectoderm

## Abstract

**Objective:**

To explore a morphometric grading system for blastocysts that is associated with ongoing pregnancy.

**Design:**

Cross-sectional study.

**Setting:**

None.

**Patients(s):**

All consecutive vitrified blastocysts at our center from July 2018 to November 2021 that were transferred in single blastocyst transfer cycles until January 2022.

**Intervention(s):**

None.

**Main Outcome Measure(s):**

The ongoing pregnancy rate after a single vitrified-warmed blastocyst transfer. Interobserver agreement on morphometric values among embryologists.

**Result(s):**

Three morphometric variables (blastocyst diameter, area of inner cell mass [ICM], and the estimated trophectoderm cell count) were used to evaluate the expansion, ICM, and trophectoderm morphology. During the study period, 585 blastocysts were involved in this study. Of the 3 morphometric variables, ICM area (per 500 μm^2^, adjusted odds ratio, 1.19; 95% confidence interval, 1.09−1.30) and estimated trophectoderm cell count (per 10 cells, adjusted odds ratio, 1.25; 95% confidence interval, 1.12−1.39) were significantly associated with the ongoing pregnancy rate after adjustment for confounding factors. The ongoing pregnancy rate was 2.0% (1/49) with an ICM area of <2,500 μm^2^ and the estimated trophectoderm cell count <70. The ongoing pregnancy rate reached 47.8% (22/46) when the ICM area and the estimated trophectoderm cell count were >3,500 μm^2^ and >110, respectively. Interobserver agreement on the blastocyst diameter, ICM area, and the estimated trophectoderm cell count was excellent-to-good among 5 embryologists (intraclass correlation coefficients: 0.99, 0.87, and 0.91, respectively).

**Conclusion(s):**

Morphometric values of ICM and trophectoderm are promising predictors of pregnancy success. The high reproducibility suggests that the morphometric variables will contribute to identifying blastocysts with the highest developmental potential as well as those that will not result in a successful pregnancy.

Identifying embryos that will be born is an important issue. Various methods have been developed to select good-quality embryos, including preimplantation genetic testing for aneuploidy, time-lapse monitoring, and omics approaches (1, 2, 3). Despite these recent advances, static morphological evaluations of embryos remain the most used methods because they are noninvasive and do not require expensive equipment. Of the several developmental stages of in vitro culture, the blastocyst morphology is considered to have the best ability to predict successful pregnancy (4, 5). Gardner and Schoolcraft established a grading system for blastocyst morphology by combining 3 morphological characteristics: degree of blastocyst expansion, size and compactness of inner cell mass (ICM), and number and cohesiveness of trophectoderm cells (6). Many studies report that the transfer of higher-grade blastocysts results in a significantly higher pregnancy rate than lower-grade blastocysts, and many fertility centers have been adopting the blastocyst morphology grading system with several modifications (7, 8, 9).

The ocular assessment of static blastocyst morphology is considered to be subjective and ambiguous (10, 11). This problem became apparent when interobserver agreement among embryologists was assessed for expansion, ICM, and trophectoderm grades in the same blastocysts. Storr et al. (12) reported that interobserver agreement on expansion, ICM, and trophectoderm grades was surprisingly low, even among experienced embryologists who attended a monthly quality assurance program. Low interobserver agreement in grading will affect the interpretation of embryo quality and data for analyzing the correlation of morphological characteristics with implantation and live birth. In fact, although many studies have explored the importance of blastocyst expansion, ICM, and trophectoderm grade, their conclusions were inconsistent (13).

Several studies have tried to develop more consistent and less subjective methods of embryo grading by quantitative measurement of blastocysts, referred to as morphometry (11). The blastocyst diameter, ICM area, and cross-sectional count of trophectoderm cells in the equatorial plane of a blastocyst are often used as morphometric variables representing the degree of expansion, ICM, and trophectoderm grade (10, 14, 15, 16). Although these studies suggested that morphometric variables were promising candidates to replace the subjective morphological assessment, their results remain inconclusive. The previous studies had several shortcomings. First, they included both fresh and frozen-thawed multiple embryo transfer (ET) cycles, which could compromise the significant influence of each blastocyst morphology on pregnancy success. Second, the cross-sectional trophectoderm cell count was insufficient to represent trophectoderm morphology because it does not reflect the total trophectoderm cell count in the blastocyst (14). Third, although the morphometric variables were considered less subjective, the reproducibility of the values was not evaluated in the previous studies.

To test the clinical usability of morphometric variables for predicting pregnancy success, we examined their relationship to the ongoing pregnancy rate in single vitrified-warmed blastocyst transfer cycles. We attempted to develop a new morphometric variable for trophectoderm, the estimated trophectoderm cell count, instead of the cross-sectional trophectoderm cell count. Because a single layer of trophectoderm cells forms the outer layer of the blastocyst just inside the zona pellucida, we estimated the trophectoderm cell count in the blastocyst as the ratio of the blastocyst surface area to the average area of trophectoderm cells (17). To assess the validity of the estimated trophectoderm cell count as a morphometric indicator of trophectoderm quality, we examined its relationship to the serum β-human chorionic gonadotropin (hCG) level after blastocyst transfer. Finally, we evaluated the reproducibility of the blastocyst diameter, ICM area, and the estimated trophectoderm cell count by calculating their interobserver agreements among embryologists.

## Materials and methods

This observational, retrospective cross-sectional study received institutional review board approval from Keio University (IRB reference number 20190091). All patients had the opportunity to opt out. To examine the relationship between morphometric values and ongoing pregnancy, this analysis included consecutive day 5 or 6 blastocysts that were vitrified at expansion stage 3 or 4 from July 2018 to November 2021 and transferred in single vitrified-warmed blastocyst transfer cycles until January 2022. Blastocysts with biopsy, blurred photographs, or an obscure trophectoderm cell boundary were excluded from the analysis. Blastocyst enrolment was terminated when the number of ongoing pregnancies reached 20 times the number of variables in the multivariate analysis.

### Ovarian Stimulation and IVF Protocol

Patients underwent ovarian stimulation by daily injection of urinary or recombinant follicle stimulating hormone with GnRH agonist or GnRH antagonist. Daily doses were adjusted according to follicle size and serum E2 levels. A dose of 5,000 IU of hCG or GnRH agonist was administered when the dominant follicle measured >17 mm, with transvaginal oocyte retrieval performed approximately 34 hours later.

Oocytes were cultured for 3−4 hours in fertilization medium (Cook) at 37.0 °C and 6.0% CO_2_ before insemination by conventional in vitro fertilization or intracytoplasmic sperm injection. Oocytes were examined 18–20 hours after insemination to determine the presence of 2 pronuclei. Fertilized oocytes were cultured in 25 μL of single-step medium (SAGE one-step medium with HSA, Origio) under oil (OVOIL, Vitrolife) at 37.0 °C, 5% O_2,_ and 6.0% CO_2_. The single-step medium was not changed during embryo culture.

### Vitrification and Warming

Blastocysts for cryopreservation were selected based on their morphological grade according to the Gardner and Schoolcraft classification. In brief, blastocysts were given scores of 1–6 based on their degree of expansion and hatching status, A–C for the morphology of ICM according to the number of cells and compactness, and A–C for the trophectoderm morphology based on the apparent number, shape, and cohesiveness of cells. Each blastocyst was graded by at least 2 experienced embryologists. When the 2 embryologists disagreed on the morphological assessment, another senior embryologist conducted the assessment.

Vitrification and warming of blastocysts were performed using commercially available kits (Cryotop open vitrification system, Kitazato) following the manufacturer’s protocol. The vitrification kits consisted of an equilibration solution (ES) and vitrification solution (VS), and the warming kits included a thawing solution (TS), diluent solution (DS), and washing solution (WS). The ES and VS contained ethylene glycol, dimethyl sulfoxide, and sucrose. For vitrification, the blastocyst was placed in ES for up to 15 minutes, followed by equilibration and dehydration for 90 seconds in VS. These solutions were maintained at room temperature during all vitrification steps. Dehydrated blastocysts were individually mounted on a Cryotop carrier, immediately placed directly into liquid nitrogen, and covered with a transparent sleeve. Vitrified blastocysts were cryopreserved in liquid nitrogen tanks until the day of the transfer to the uterus.

Blastocysts were warmed for 3−4 hours before ET. In brief, a Cryotop carrier was pulled from the sleeve, and the liquid nitrogen, and immediately immersed in TS at 37 °C. One minute later, the blastocyst was moved into DS and incubated for 3 minutes. Then, the blastocyst was placed in WS for 5–10 minutes. The incubation steps from DS to WS were performed at room temperature. Assisted hatching was performed by opening a 50 μm hole in the zona pellucida using a laser system (Octax Navilase, Vitrolife). Warmed blastocysts were cultured in a SAGE one-step medium for at least 2 hours, and blastocoel recovery was checked before transfer into the uterus.

### Embryo Transfer

Embryo transfer was performed under transabdominal or transvaginal ultrasound guidance in cycles with spontaneous ovulation or hormone replacement treatment. Clinical pregnancies were diagnosed by the presence of a gestational sac on transvaginal ultrasound approximately 2 weeks after transfer.

### Morphometric Assessment of Blastocyst

Blastocysts were observed at 400× magnification at 116±2 hours after insemination for day 5 blastocysts and 140±2 hours for day 6 blastocysts using an inverted microscope (IX71, Olympus). Three digital images were taken for each blastocyst as a part of routine practice using Cronos 3 through a charge coupled device camera (CS230B, Olympus). One image focused on the equatorial plane of the blastocyst; the 2 others focused on trophectoderm cells at the top and bottom sides of the blastocyst. If the ICM was out of focus in any of these 3 images, an additional image was taken with the ICM in focus.

Blastocysts were measured using the straight line or polygon tool in Image J software (ver.1.52). The blastocyst diameter was calculated as the average of 2 orthogonal diameters in the equatorial plane of the blastocyst. We did not include the zona pellucida in the diameter measurements. The ICM area was measured by encircling ICM using a polygon tool in the software program (14). The blastocyst surface area was calculated by quadrupling the area of the equatorial plane of the blastocyst. To measure the trophectoderm cell area, we selected at least 5 trophectoderm cells that were in focus and traced the contour of the cells using the polygon tool. Then, the average trophectoderm cell area was calculated as the trophectoderm cell area. The same embryologist (H.U.) measured all blastocysts without knowing the results of ET.

### Interobserver Agreement on Morphometric Values

Interobserver agreement on 3 morphometric values was estimated by calculating the intraclass correlation coefficient (ICC) for 5 embryologists. All 5 embryologists practiced at different facilities, with 15, 14, 13, 8, and 4 years of clinical experience. They measured the 3 morphometric variables (blastocyst diameter, ICM area, and the estimated trophectoderm cell count) in the same 100 blastocysts. Embryologists were blinded to the measurements of other embryologists. The ICC estimates and their 95% confidence intervals were calculated using the psych library in R 4.1.2. based on a single-rating, absolute-agreement, two-way random-effects model. H.U. instructed each embryologist on the measuring procedure in at least 3 blastocysts. The ICC values of <0.5, 0.5–0.75, 0.75–0.9, and >0.9 indicated poor, moderate, good, and excellent interobserver agreement, respectively (18).

### Association Between Morphometric Values and Pregnancy Success

A pregnancy that continued until the second trimester was considered an ongoing pregnancy. Because some couples transferred blastocysts more than once within the study period, a generalized estimating equation (GEE) analysis was used to analyze the effects of possible explanatory variables on ongoing pregnancy. The following possible explanatory variables were included in our multivariate GEE model: woman’s age, number of previous oocyte pick-up (OPU) cycles, number of previous ET cycles, assisted hatching, blastocyst day, blastocyst diameter, ICM area, and estimated trophectoderm cell count. Blastocyst diameter, ICM area, and estimated trophectoderm cell count were rounded to the nearest 10 μm, 500 μm^2^, and 10 cells, respectively. These cutoff points were determined according to their standard deviations (2.2, 281, and 8.0, respectively). Each explanatory variable was also analyzed in a univariate GEE model.

The relationship between estimated trophectoderm cell count and serum β-hCG after blastocyst transfer was examined. For this analysis, 88 blastocyst transfers were included, and serum β-hCG was measured 9, 10, or 11 days after transfer. We used a generalized linear model including 2 explanatory variables: estimated trophectoderm cell count and the number of days after blastocyst transfer.

All statistical analyses were performed using R 4.1.2. Mean and median values were accompanied by standard deviation and interquartile range (IQR), respectively. Two-tailed *P*-values of <.05 were considered statistically significant.

## Results

During the study period, 673 consecutive single vitrified-warmed blastocyst transfers met our inclusion criteria; 19, 30, and 38 were excluded because of suboptimal photographs, ambiguous boundaries between trophectoderm cells, and biopsy, respectively. Thus, 585 transfers in 299 couples were included. Of these, 491 blastocysts were transferred on day 5; the remaining 94 were transferred on day 6. The average woman’s age at oocyte retrieval was 37.2±3.9 years. The medians of previous OPU and embryo cycles were 0 (IQR: 0–1) and 1 (IQR: 0–3) cycles, respectively. Assisted hatching took place for 168 blastocysts. The average blastocyst diameter, ICM area, and estimated trophectoderm cell count were 170±23 μm, 3,014±1,071 μm^2^, and 93±32, respectively. The blastocyst diameter was significantly correlated with both the ICM area (r=0.132, *P*=.001) and the estimated trophectoderm cell count (r=0.533, *P*<.001) (Supplemental Fig.1, available online). The ICM area was not correlated with the estimated trophectoderm cell count (Supplemental Fig.1).

The univariate GEE analyses showed that the woman’s age at OPU (years, crude odds ratio [OR] 0.88; 95% confidence interval [CI], 0.84−0.93, *P<*.001), number of previous OPU cycles (crude OR 0.76; 95% CI, 0.63−0.92, *P=*.004), number of previous ET cycles (crude OR 0.82; 95% CI, 0.73−0.92, *P<*.001), blastocyst day (day 5 vs. 6, crude OR 0.43; 95% CI, 0.22−0.85, *P=*.015), blastocyst diameter (per 10 μm, crude OR 1.10; 95% CI, 1.01−1.19, *P=*.02), ICM area (per 500 μm^2^, crude OR 1.20; 95% CI, 1.10−1.30, *P<*.001), and estimated trophectoderm cell count (per 10 cells, crude OR 1.18; 95% CI, 1.09−1.27, *P<*.001) were significantly associated with the ongoing pregnancy rate before adjustment for confounding factors (Table 1).

**Table 1:** Univariate and multivariate generalized estimating equation analyses of factors associated with ongoing pregnancy rate.

	Univariate analysis		Multivariate analysis	
	OR (95% CI)	*P* value	OR (95% CI)	*P* value
Women’s age (y)	0.88 (0.84-0.93)	< .001	0.91 (0.86-0.96)	.001
No. of previous OPU cycles	0.76 (0.63-0.92)	.004	0.93 (0.76-1.13)	.466
No. of previous ET cycles	0.82 (0.73-0.92)	<.001	0.90 (0.80-1.01)	.081
Assisted hatching	0.69 (0.43-1.09)	.112	0.81 (0.49-1.34)	.416
Day				
5	reference		reference	
6	0.43 (0.22-0.85)	.015	0.33 (0.14-0.79)	.013
Blastocyst diameter (per 10 μm)	1.10 (1.01-1.19)	.02	0.92 (0.82-1.04)	.177
ICM area (per 500 μm^2^)	1.20 (1.10-1.30)	<.001	1.19 (1.09-1.30)	<.001
Estimated trophectoderm cell count (per 10 cells)	1.18 (1.09-1.27)	<.001	1.24 (1.11-1.38)	<.001

CI = confidence interval; ET = embryo transfer; ICM = inner cell mass; OPU = oocyte pick-up.

In the multivariate GEE analysis, woman’s age at OPU (years, adjusted OR 0.91; 95% CI, 0.86−0.96, *P=*.001), blastocyst day (day 5 vs. 6, adjusted OR 0.30; 95% CI, 0.13−0.72, *P=*.008), ICM area (per 500 μm^2^, adjusted OR 1.19; 95% CI, 1.09−1.30, *P<*.001), estimated trophectoderm cell count (per 10 cells, adjusted OR 1.25; 95% CI, 1.12−1.39, *P<*.001) remained as significant independent predictors of ongoing pregnancy (Table 1). The number of previous OPU cycles, previous ET cycles, and blastocyst diameter did not retain significance.

A predictive model with the significant variables was constructed to calculate the probability of ongoing pregnancy with the equation: Expectedongoingpregnancyrate=1/[1+exp(−6.5405+0.1077×femaleage+1.2931×blastocystday−0.1647×ICMarea/500−trophectodermcellcount/10)]. To examine the effect of this model on embryo selection, we calculated the expected ongoing pregnancy rate for 128 patients who underwent at least 2 SETs during the study period. We found that 45% (57/128) of patients received a blastocyst with a higher expected ongoing pregnancy rate in the second SET than in the first SET. Of these, 25% (14/57) patients did not have an ongoing pregnancy after the first SET but reached it after the second SET. Furthermore, no patient achieved ongoing pregnancy after the first SET and failed pregnancy after the second one among the 57 patients.

**Figure 1:**
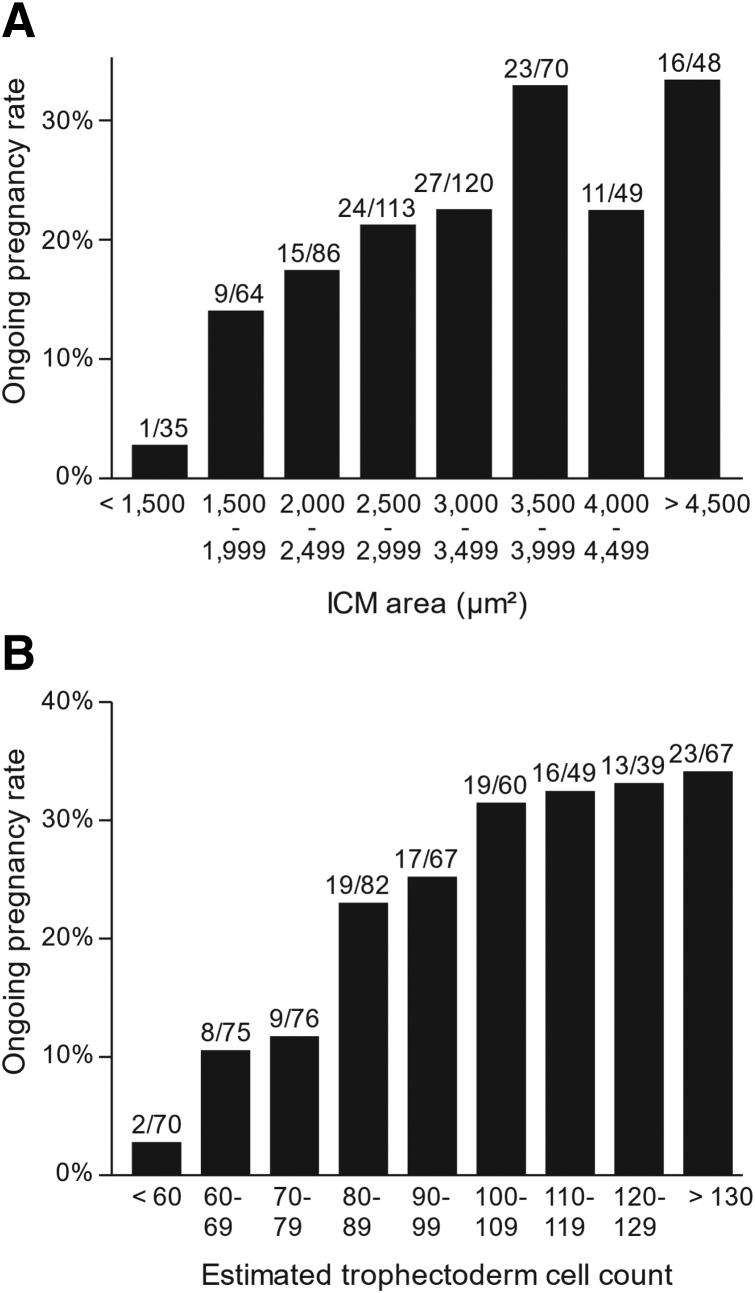
The effects of inner cell mass (ICM) area **(A)** and the estimated trophectoderm cell count **(B)** on ongoing pregnancy rate. The numerator and denominator of each bar show the number of blastocysts that reached ongoing pregnancy and the number of blastocysts transferred, respectively.

Figure 1 shows the relationship between the statistically significant morphometric variables and the ongoing pregnancy rate. The ongoing pregnancy rate was 2.9% (1/35) with an ICM area of <1,500 μm^2^, whereas it reached 33.3% (16/48) with an ICM area of >4,500 μm^2^. With respect to the estimated trophectoderm cell count, the ongoing pregnancy rate was 2.9% (2/70) when the cell count was <60. The ongoing pregnancy rate consistently increased with the number of cells, approaching 34.3% (23/67) with a cell count of >130.

Because the ICM area and the estimated trophectoderm cell count were independently associated with ongoing pregnancy, we calculated their combined effects on ongoing pregnancy (Table 2). The ongoing pregnancy rate was 2.0% (1/49) with an ICM area of <2,500 μm^2^ and the estimated trophectoderm cell count <70. The rate reached 47.8% (22/46) with an ICM area of >3,500 μm^2^ and an estimated trophectoderm cell count >110.

**Table 2:** The combined effects of inner cell mass (ICM) area and the estimated trophectoderm cell count on the ongoing pregnancy rate.

ICM area (μm^2^)	Estimated trophectoderm cell count			
	< 70	70–89	90–109	≥ 110
< 2,500 (%)	2.0 (1/49)	18.5 (10/54)	21.6 (8/37)	13.3 (6/45)
2,500–2,999 (%)	7.1 (2/28)	17.4 (4/23)	17.2 (5/29)	39.4 (13/33)
3,000–3,499 (%)	10.0 (3/30)	14.3 (5/35)	33.3 (8/24)	35.5 (11/31)
≥ 3,500 (%)	10.5 (4/38)	19.6 (9/46)	40.5 (15/37)	47.8 (22/46)

*Note:* The cutoff values were determined so that the denominators were >20.

To test the assumption that the estimated trophectoderm cell count reflected the developmental potential of trophectoderm, we analyzed its relationship to the serum β-hCG level at 9, 10, or 11 days after ET that reached a serum β-hCG level of >10 mIU/ml at measurement. A multivariate generalized linear model analysis showed that the estimated trophectoderm cell count was significantly associated with serum hCG (*P<*.005). Serum hCG increased with the estimated trophectoderm cell count (Supplemental Fig.2).

**Figure 2:**
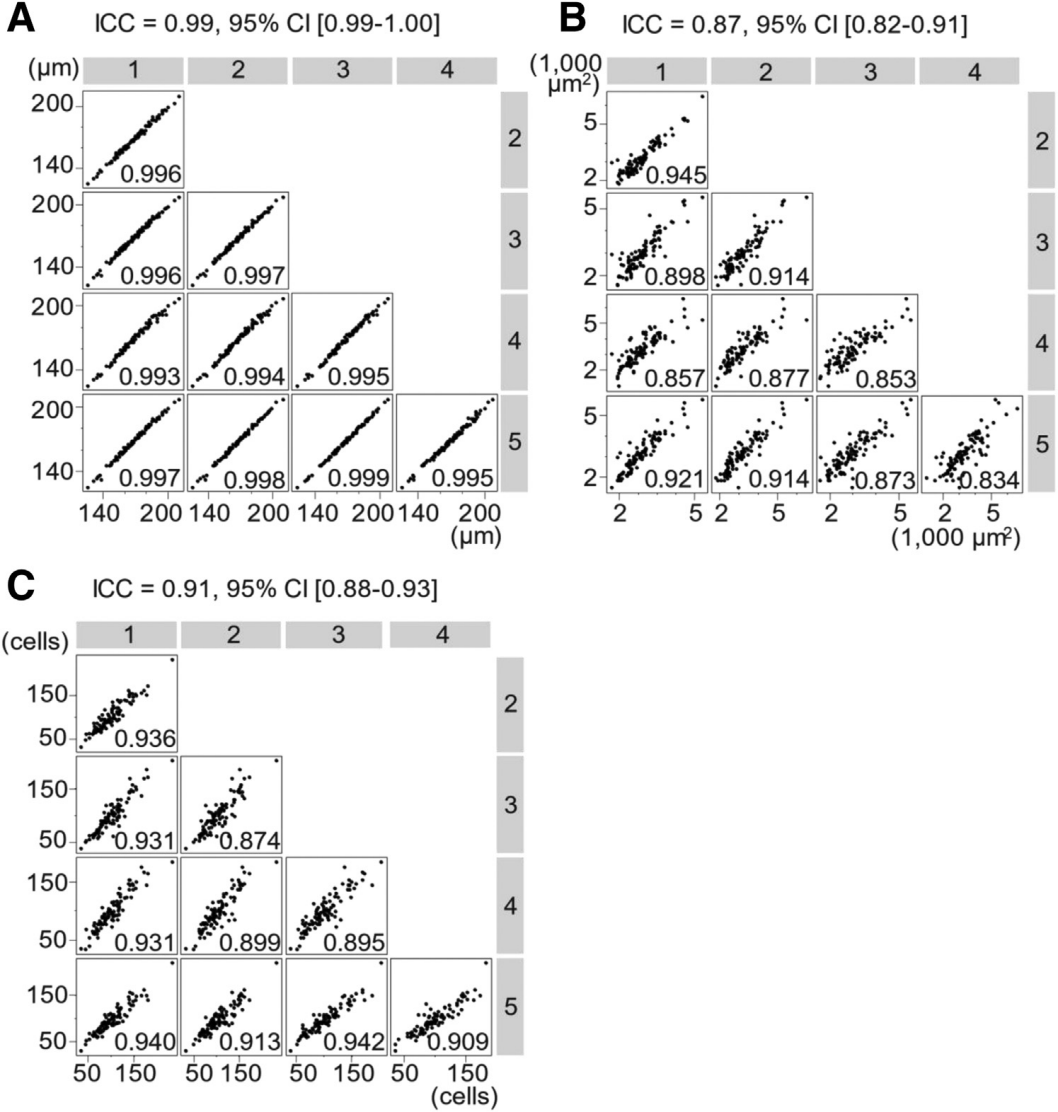
The correlation of morphometric measurements among 5 embryologists. Numeric characters in gray boxes indicate the embryologists. The correlation coefficient is shown in each box. **A,** Blastocyst diameter; **B,** Inner cell mass (ICM) area; **C,** The estimated trophectoderm cell count. ICC = intraclass correlation coefficient; CI = confidence interval.

The intraclass correlation coefficients for blastocyst diameter, ICM area, and the estimated trophectoderm cell count were 0.99 (95% confidence interval [CI], 0.99−1.00), 0.87 (95% CI, 0.82−0.91), and 0.91 (95% CI, 0.88−0.93), respectively (Fig. 2). These ICC values indicated that the interobserver agreements on the blastocyst diameter, ICM area, and the estimated trophectoderm cell count were excellent, good-to-excellent, and good-to-excellent, respectively.

We also examined the relationship between the morphometric values and conventional grades of blastocyst morphology. The blastocyst diameter, ICM area, and estimated trophectoderm cell count were significantly correlated with the expansion (r=0.74, *P*<.001), ICM (r=0.38, *P*<.001), and trophectoderm (r=0.45, *P*<.001) grades, respectively. However, the ranges of the morphometric variables overlapped between different morphological grades (Supplemental Fig.3). Univariate GEE analyses demonstrated that the conventional morphology grades were significantly associated with the ongoing pregnancy rate in the absence of morphometric variables (expansion stage 3 vs. 4, crude OR 2.06; 95% CI, 1.25−3.40, *P=*.004; ICM grade A vs. B, crude OR 0.50; 95% CI, 0.37−0.75, *P<*.001; TE grade A vs. B, crude OR 0.53; 95% CI, 0.35−0.81, *P<*.001) (Supplemental Fig.4; Supplemental Table 1, available online). However, the effects of the conventional morphological grades on ongoing pregnancy disappeared in a multivariate GEE model that included both morphometric variables and conventional morphological grades (expansion stage 3 vs. 4, adjusted OR 1.73; 95% CI, 0.85−3.52, *P=*.13; ICM grade A vs. B or C, adjusted OR 0.71; 95% CI, 0.45−1.12, *P=*.14; TE grade A vs. B or C, adjusted OR 0.73; 95% CI, 0.45−1.17, *P=*.19) (Supplemental Table 1).

## Discussion

The current study demonstrated that the ICM area and the estimated trophectoderm cell count were both significant predictors of ongoing pregnancy reaching the second trimester. When the 2 morphometric variables were assessed simultaneously, the ongoing pregnancy rate was 47.8% (22/46) in blastocysts with ICM >2,500 μm^2^ and the estimated trophectoderm cell count >110, whereas it was only 2.0% (1/47) in blastocysts with ICM <2,500 μm^2^ and estimated trophectoderm cell count <70. The morphometric values showed excellent-to-good interobserver agreement, suggesting high reproducibility among embryologists.

Morphometric assessment of blastocyst morphology has been explored in several studies. Richter et al. (10) showed that the implantation rate was higher among blastocysts with an ICM area of >4,500 μm^2^ (45%), whereas an ICM area of <3,800 μm^2^ was associated with a low implantation rate (18%). Although their results were consistent with ours, their method overestimated the actual ICM area (14). As a result, the influence of the ICM area on pregnancy success demonstrated in their findings cannot be compared with ours. A more recent study measured 254 blastocysts and argued that the cross-sectional trophectoderm cell count was associated with implantation success and live birth but failed to find an association of the quantified ICM size with live birth (14). This discrepancy in relation to our results may be attributable to the difference in the distributions of the ICM area between the previous and the present studies (3,702±1,216 μm^2^ and 3,014±1,071 μm^2^, respectively). Our results suggested that the relationship between the ICM area and the ongoing pregnancy rate was weak in blastocysts with a large ICM area (>3,500 μm^2^) (Fig.1).

In contrast to our results, several studies showed that blastocyst diameter was significantly associated with a successful pregnancy after blastocyst transfer (15, 16). There are 2 possible reasons for the contradiction. First, the present study did not involve fresh blastocyst transfer cycles. Therefore, blastocyst diameter was suggested to be a more important variable in fresh blastocyst transfer cycles than in vitrified-warmed blastocyst transfer cycles because the endometrial advancement in fresh cycles would favor the fastest developing blastocyst from the viewpoint of synchrony between the embryo and endometrium. Second, the blastocyst diameter was significantly correlated with the estimated trophectoderm cell count. Thus, the effect of the estimated trophectoderm cell count would offset the effect of blastocyst diameter on ongoing pregnancy in the multivariate analysis.

In addition to blastocyst diameter and ICM area, we developed a new morphometric variable for trophectoderm morphology, the estimated trophectoderm cell count, by calculating the ratio of blastocyst surface area to the trophectoderm cell area. The trophectoderm cell count has possible advantages over the cross-sectional trophectoderm cell count in the equatorial plane used in previous studies. First, the wide range of the estimated trophectoderm cell count will increase the discriminatory power of trophectoderm morphology. Second, the estimated trophectoderm cell count was significantly associated with the serum β-hCG level after blastocyst transfer. This is the first study to show an association between trophectoderm morphology and serum β-hCG. Given that the serum β-hCG is secreted by invasive trophoblasts, this association supports our findings that the estimated trophectoderm cell count reflects the implantation potential of blastocysts.

The strengths of morphometric assessment of blastocysts are noninvasiveness and cost-effectiveness, as it is performed using digital images taken in routine static embryo observations. Furthermore, the interobserver agreements of the 3 morphometric variables obtained in the current study were surprisingly high compared with the conventional morphological grades in previous studies (12, 19). Therefore, the morphometric approach may improve the predictive ability of blastocyst morphology for pregnancy success. Indeed, the effects of conventional morphological grades on ongoing pregnancy disappeared in the multivariate GEE analysis that included both conventional and 3 morphometric variables (Supplemental Table 1), indicating that the morphometric assessment was more likely to predict the ongoing pregnancy rate than the conventional morphological grades. Furthermore, our results suggested that if blastocysts had been selected based on the morphometric assessments, a different embryo would have been selected at the first SET in 45% of patients, which could have contributed to reducing the time to pregnancy in 14 patients during the study period. Also, the high interobserver agreement suggested that the morphometric approach would be useful for comparing the effects of blastocyst morphology on successful pregnancy among different facilities.

Our multivariate analysis suggested that the ongoing pregnancy rate after vitrified-warmed blastocyst transfer of day 6 blastocysts was significantly lower than that of day 5 blastocysts, regardless of their morphometric values. This result concords with previous studies that reported the significant negative effects of delayed development on live birth in frozen-thawed ET cycles (20, 21, 22). The lower ongoing pregnancy rate with day 6 blastocysts may be attributable to the higher aneuploidy rate in delayed blastulation (23, 24, 25). However, the chromosomal factor may not solely explain the difference in pregnancy rate because day 5 and 6 euploid blastocysts still showed different implantation potential, even with similar morphological grades (26). Future studies regarding our morphometric variables may provide new insight into the relationship between ploidy, blastulation time, and blastocyst morphology (27).

The present study was associated with some limitations. First, the retrospective nature of this study does not guarantee the usefulness of the morphometric values in the selection of embryos before ET into the uterus. Second, there is room for improvement in ICM morphometry. Because the ICM is a three-dimensional dynamic structure (28), a two-dimensional area would not accurately reflect the ICM volume. Estimating the ICM volume may further increase the predictive ability of ICM morphology for the pregnancy outcome. Third, the association between pregnancy success and the morphometric variables at times other than 116±2 hours and 140±2 hours remains unclear. Further studies are needed to investigate the effects of time after insemination in addition to the morphometric variables using a time-lapse imaging system.

In conclusion, ICM morphometry and the estimated trophectoderm cell count are promising predictors of pregnancy success. The morphometric grading system is expected to reduce interobserver and interinstitutional variability in the morphological assessment of blastocysts, which will contribute to the identification of blastocysts with the highest developmental potential and reduce the time to pregnancy. Future prospective studies are needed to determine the effect of the combination of the ICM area and the estimated trophectoderm cell count on successful pregnancy. The present results could be used as a training set for confirming the usefulness of the morphometric assessment.

## Supplementary data

Supplemental figure 1 The relationship between blastocyst diameter, inner cell mass (ICM) area, and the estimated trophectoderm cell count. Each plot represents an embryo.

Supplemental figure 2 The relationship between the estimated trophectoderm cell count and serum β-hCG level after blastocyst transfer. Filled circles, open circles, and open squares indicate the serum β-hCG levels at 9, 10, and 11 days after embryo transfer. Each plot represents a different embryo transfer.

Supplemental figure 3 The association between the degree of expansion and blastocyst diameter (A), the inner cell mass (ICM) grade and ICM area (B), and the estimated trophectoderm cell count (C). The degree of expansion, ICM grade, and trophectoderm grade were evaluated using the Gardner and Schoolcraft classification system.

Supplemental figure 4 Ongoing pregnancy rate and blastocyst morphology grades according to the Gardner & Schoolcraft classification. The denominator and numerator of each bar show the number of blastocysts that reached ongoing pregnancy and the number of blastocysts transferred, respectively.

## References

[1] Munné S., Kaplan B., Frattarelli J.L., Child T., Nakhuda G., Shamma F.N. (2019). Preimplantation genetic testing for aneuploidy versus morphology as selection criteria for single frozen-thawed embryo transfer in good-prognosis patients: a multicenter randomized clinical trial. Fertil Steril.

[2] Sciorio R., Meseguer M. (2021). Focus on time-lapse analysis: blastocyst collapse and morphometric assessment as new features of embryo viability. Reprod Biomed Online.

[3] Bracewell-Milnes T., Saso S., Abdalla H., Nikolau D., Norman-Taylor J., Johnson M. (2017). Metabolomics as a tool to identify biomarkers to predict and improve outcomes in reproductive medicine: a systematic review. Hum Reprod Update.

[4] Papanikolaou E.G., Camus M., Kolibianakis E.M., Van Landuyt L., Van Steirteghem A., Devroey P. (2006). In vitro fertilization with single blastocyst-stage versus single cleavage-stage embryos. Obstet Gynecol Surv.

[5] Glujovsky D., Quinteiro Retamar A.M., Alvarez Sedo C.R., Ciapponi A., Cornelisse S., Blake D. (2022). Cleavage-stage versus blastocyst-stage embryo transfer in assisted reproductive technology. Cochrane database Syst Rev.

[6] Gardner D.K., Schoolcraft W.B., Jansen R., Mortimer D. (1999). Towards reproductive certainty: fertility and genetics beyond 1999.

[7] Van den Abbeel E., Balaban B., Ziebe S., Lundin K., Cuesta M.J.G., Klein B.M. (2013). Association between blastocyst morphology and outcome of single-blastocyst transfer. Reprod Biomed Online.

[8] Balaban B., Urman B., Sertac A., Alatas C., Aksoy S., Mercan R. (2000). Blastocyst quality affects the success of blastocyst-stage embryo transfer. Fertil Steril.

[9] Balaban B., Brison D., Calderón G., Catt J., Conaghan J., Cowan L. (2011). Istanbul consensus workshop on embryo assessment: proceedings of an expert meeting. Hum Reprod.

[10] Richter K.S., Harris D.C., Daneshmand S.T., Shapiro B.S. (2001). Quantitative grading of a human blastocyst: optimal inner cell mass size and shape. Fertil Steril.

[11] Lundin K., Ahlström A. (2015). Quality control and standardization of embryo morphology scoring and viability markers. Reprod Biomed Online.

[12] Storr A., Venetis C.A., Cooke S., Kilani S., Ledger W. (2017). Inter-observer and intra-observer agreement between embryologists during selection of a single day 5 embryo for transfer: a multicenter study. Hum Reprod.

[13] Morbeck D.E. (2017). Blastocyst culture in the era of PGS and freezealls: is a ‘C’ a failing grade?. Hum Reprod Open.

[14] Ebner T., Tritscher K., Mayer R.B., Oppelt P., Duba H.C., Maurer M. (2016). Quantitative and qualitative trophectoderm grading allows for prediction of live birth and gender. J Assist Reprod Genet.

[15] Shapiro B.S., Daneshmand S.T., Garner F.C., Aguirre M., Thomas S. (2008). Large blastocyst diameter, early blastulation, and low preovulatory serum progesterone are dominant predictors of clinical pregnancy in fresh autologous cycles. Fertil Steril.

[16] Sciorio R., Thong D., Thong K.J., Pickering S.J. (2021). Clinical pregnancy is significantly associated with the blastocyst width and area: a time-lapse study. J Assist Reprod Genet.

[17] Jones RE, Lopez KH. Human reproductive biology. 3rd ed. Elsevier Academic Press; 2006.

[18] Koo T.K., Li M.Y. (2016). A guideline of selecting and reporting intraclass correlation coefficients for reliability research. J Chiropr Med.

[19] Cimadomo D., Sosa Fernandez L., Soscia D., Fabozzi G., Benini F., Cesana A. (2022). Inter-centre reliability in embryo grading across several IVF clinics is limited: implications for embryo selection. Reprod Biomed Online.

[20] Ferreux L., Bourdon M., Sallem A., Santulli P., Barraud-Lange V., Le Foll N. (2018). Live birth rate following frozen–thawed blastocyst transfer is higher with blastocysts expanded on day 5 than on day 6. Hum Reprod.

[21] Haas J., Meriano J., Laskin C., Bentov Y., Barzilay E., Casper R.F. (2016). Clinical pregnancy rate following frozen embryo transfer is higher with blastocysts vitrified on day 5 than on day 6. J Assist Reprod Genet.

[22] Bourdon M., Pocate-Cheriet K., Finet De Bantel A., Grzegorczyk-Martin V., Amar Hoffet A., Arbo E. (2019). Day 5 versus day 6 blastocyst transfers: a systematic review and meta-analysis of clinical outcomes. Hum Reprod.

[23] Taylor T.H., Patrick J.L., Gitlin S.A., Wilson J.M., Crain J.L., Griffin D.K. (2014). Comparison of aneuploidy, pregnancy and live birth rates between day 5 and day 6 blastocysts. Reprod Biomed Online.

[24] Kaing A., Kroener L.L., Tassin R., Li M., Liu L., Buyalos R. (2018). Earlier day of blastocyst development is predictive of embryonic euploidy across all ages: essential data for physician decision-making and counseling patients. J Assist Reprod Genet.

[25] Tong J., Niu Y., Wan A., Zhang T. (2022). Comparison of day 5 blastocyst with day 6 blastocyst: evidence from NGS-based PGT-A results. J Assist Reprod Genet.

[26] Irani M., Reichman D., Robles A., Melnick A., Davis O., Zaninovic N. (2017). Morphologic grading of euploid blastocysts influences implantation and ongoing pregnancy rates. Fertil Steril.

[27] Huang T.T., Huang D.H., Ahn H.J., Arnett C., Huang C.T. (2019). Early blastocyst expansion in euploid and aneuploid human embryos: evidence for a non-invasive and quantitative marker for embryo selection. Reprod Biomed Online.

[28] Kirkegaard K., Agerholm I.E., Ingerslev H.J. (2012). Time-lapse monitoring as a tool for clinical embryo assessment. Hum Reprod.

