# Supplementary figures and images for: Morphometric assessment of blastocysts: relationship with the ongoing pregnancy rate
Source: F S Rep. 2022 Nov 11;4(1):85–92. doi: 10.1016/j.xfre.2022.11.001 (PMC10028418; doi:10.1016/j.xfre.2022.11.001)

## Slide 1
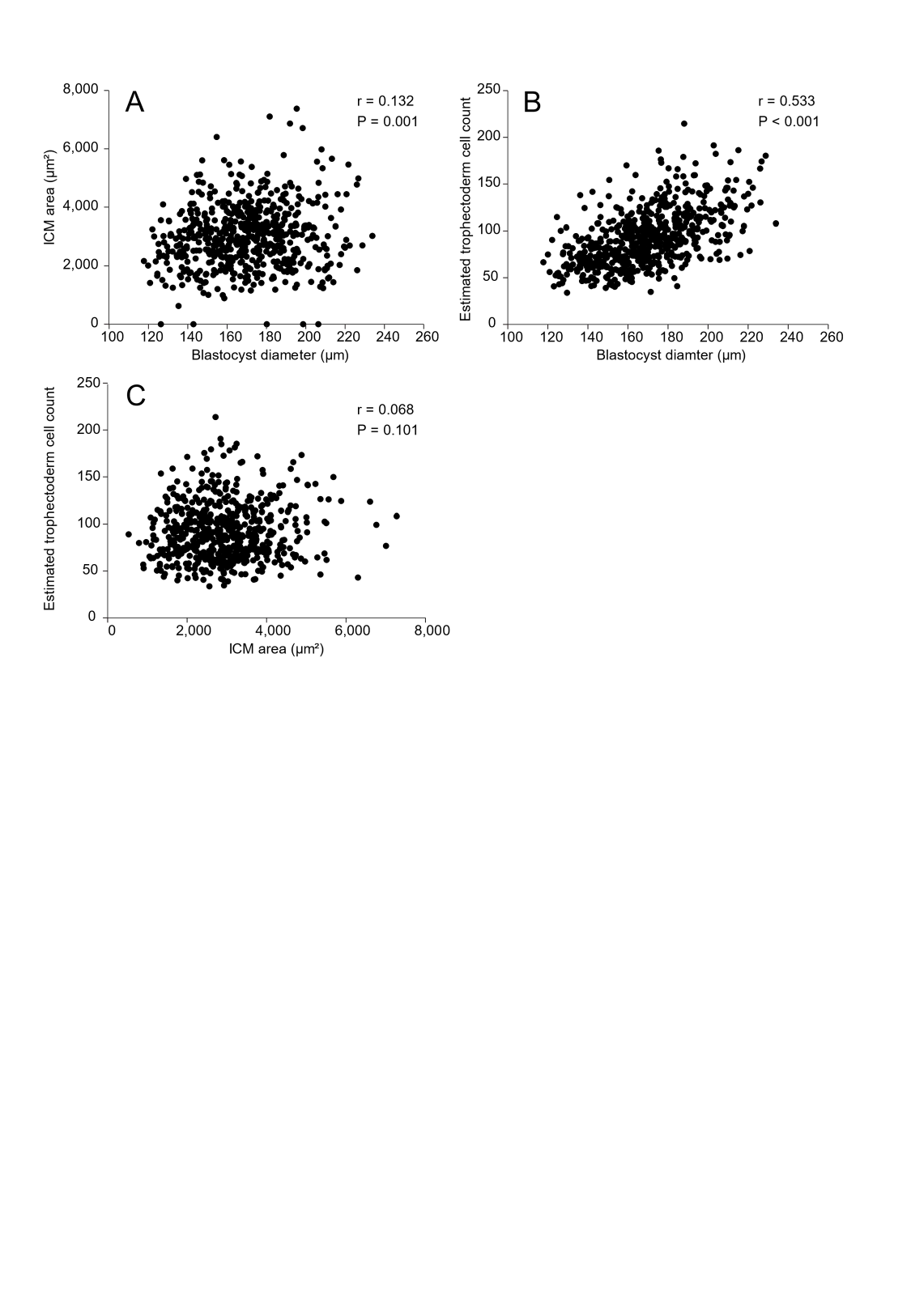

Supplement: Supplemental figure 1 — The relationship between blastocyst diameter, inner cell mass (ICM) area, and the estimated trophectoderm cell count. Each plot represents an embryo. [file mmc1.ppt]

## Slide 1
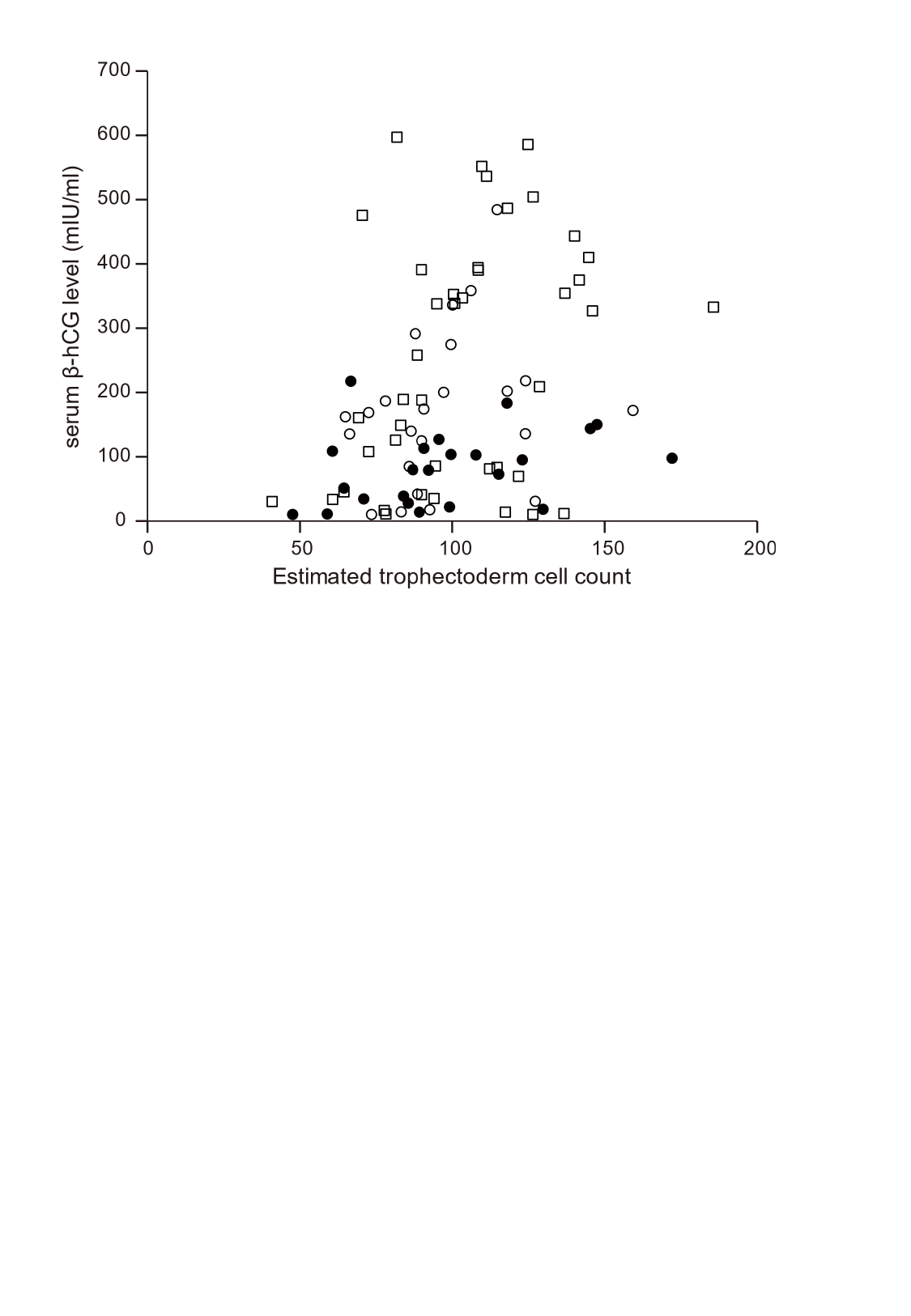

Supplement: Supplemental figure 2 — The relationship between the estimated trophectoderm cell count and serum β-hCG level after blastocyst transfer. Filled circles, open circles, and open squares indicate the serum β-hCG levels at 9, 10, and 11 days after embryo transfer. Each plot represents a different embryo transfer. [file mmc2.ppt]

## Slide 1
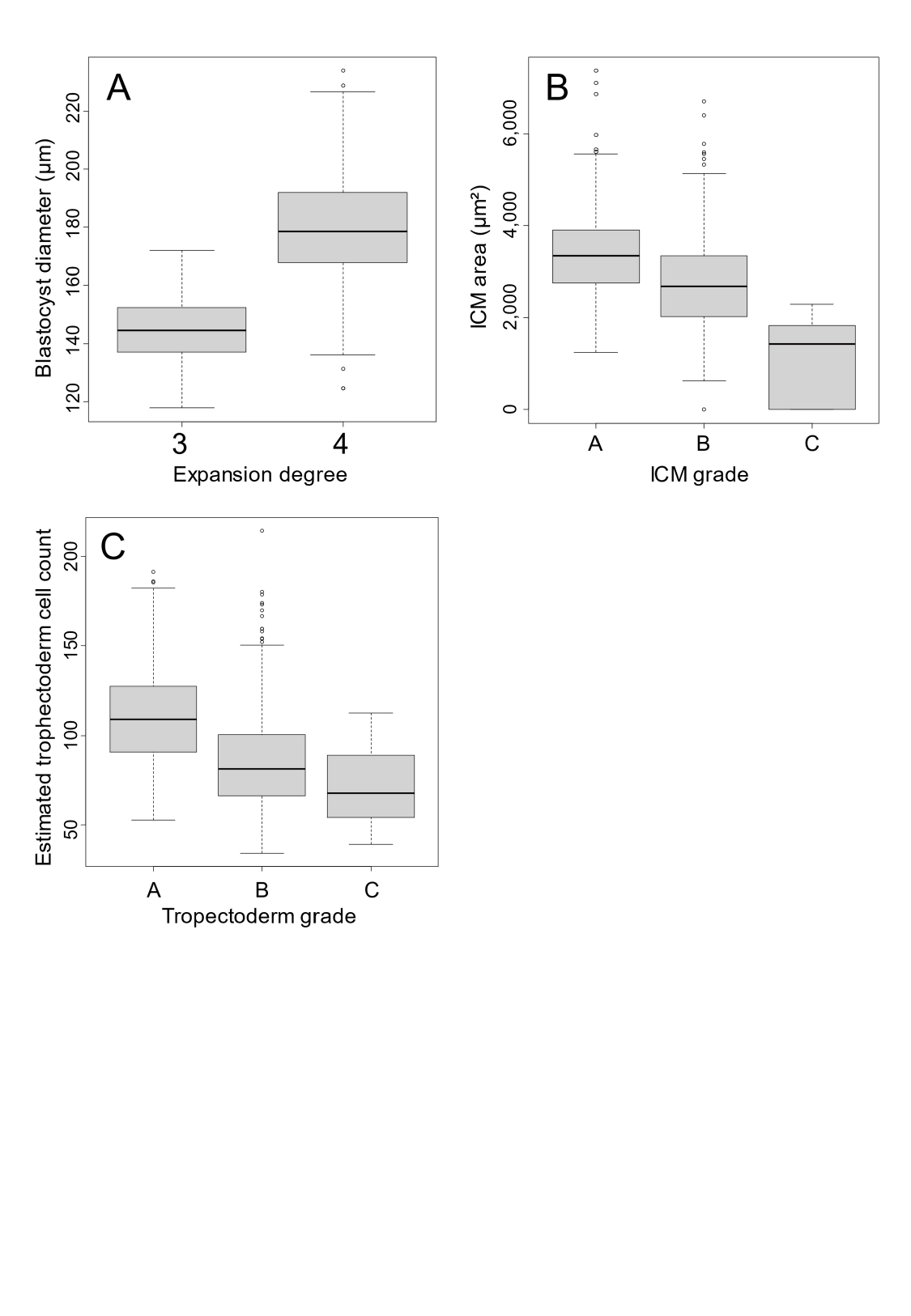

Supplement: Supplemental figure 3 — The association between the degree of expansion and blastocyst diameter (A), the inner cell mass (ICM) grade and ICM area (B), and the estimated trophectoderm cell count (C). The degree of expansion, ICM grade, and trophectoderm grade were evaluated using the Gardner and Schoolcraft classification system. [file mmc3.ppt]

## Slide 1
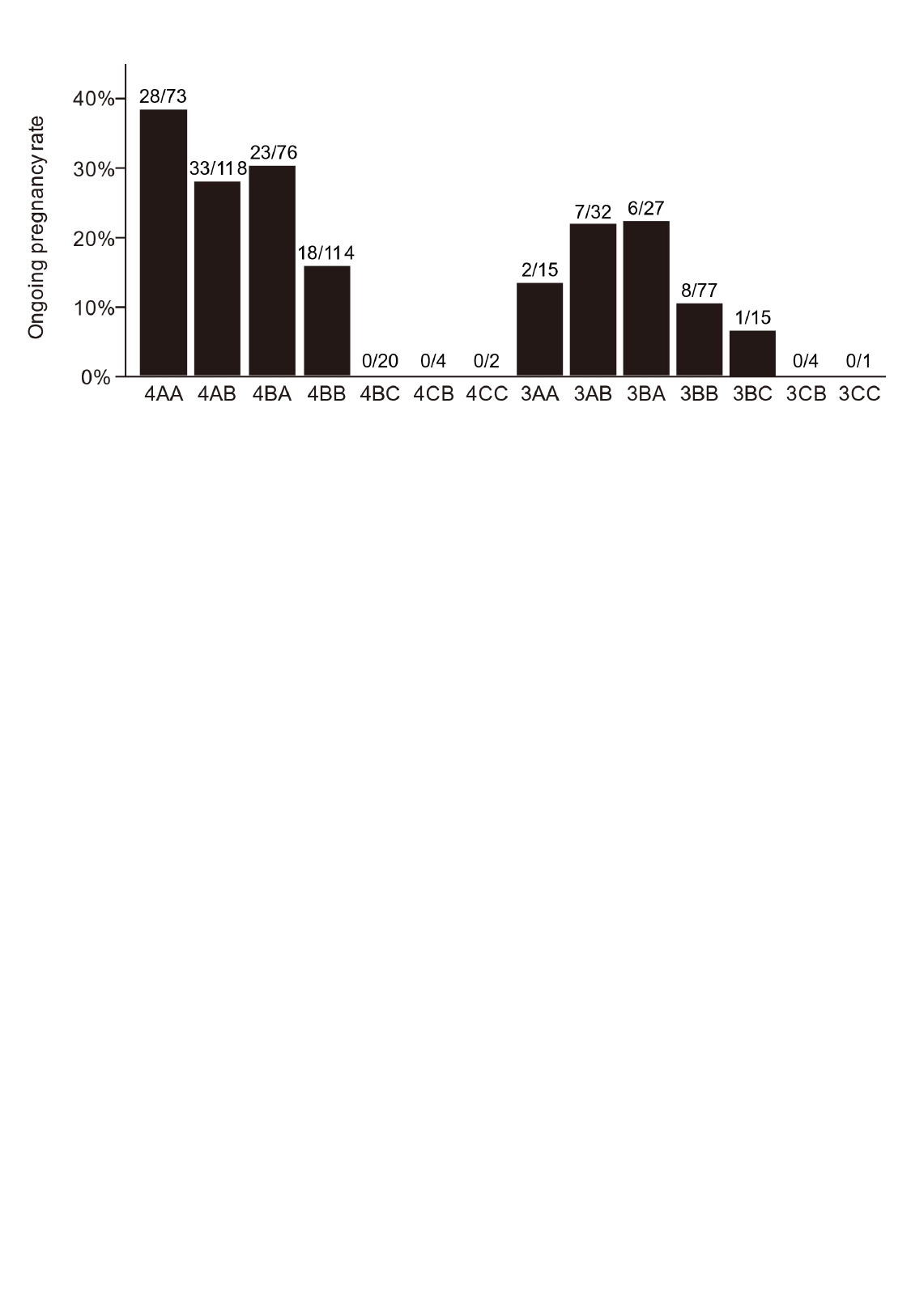

Supplement: Supplemental figure 4 — Ongoing pregnancy rate and blastocyst morphology grades according to the Gardner & Schoolcraft classification. The denominator and numerator of each bar show the number of blastocysts that reached ongoing pregnancy and the number of blastocysts transferred, respectively. [file mmc4.ppt]
