# Supplementary material for: Morphometric assessment of blastocysts: relationship with the ongoing pregnancy rate
Source: F S Rep. 2022 Nov 11;4(1):85–92. doi: 10.1016/j.xfre.2022.11.001 (PMC10028418; doi:10.1016/j.xfre.2022.11.001)
Supplement: Supplemental table 1 [file mmc5.docx]

Supplemental table 1. Univariate and multivariate GEE analyses including conventional morphological grades according to the Gardner and Schoolcraft classification.

|  | Univariate analysis | | Multivariate analysis | |
| --- | --- | --- | --- | --- |
|  | OR (95% CI) | P-value | OR (95% CI) | P-value |
| Female age (year) | 0.88 (0.84-0.93) | < 0.001 | 0.91 (0.87-0.97) | 0.002 |
| Number of previous OPU cycles | 0.76 (0.63-0.92) | 0.004 | 0.92 (0.75-1.13) | 0.421 |
| Number of previous ET cycles | 0.82 (0.73-0.92) | < 0.001 | 0.92 (0.81-1.03) | 0.143 |
| Assisted hatching | 0.69 (0.43-1.09) | 0.112 | 0.81 (0.50-1.34) | 0.420 |
| Day |  |  |  |  |
| 5 | reference |  | reference |  |
| 6 | 0.43 (0.22-0.85) | 0.015 | 0.42 (0.17-1.02) | 0.056 |
| Blastocyst diameter (per 10 μm) | 1.10 (1.01-1.19) | 0.02 | 0.87 (0.75-1.01) | 0.051 |
| ICM area (per 500 μm^2^) | 1.20 (1.10-1.30) | < 0.001 | 1.16 (1.05-1.28) | 0.003 |
| Estimated trophectoderm cell count (per 10 cells) | 1.18 (1.09-1.27) | < 0.001 | 1.20 (1.07-1.36) | 0.003 |
| Expansion stage |  |  |  |  |
| 3 | reference |  | reference |  |
| 4 | 2.06 (1.25-3.04) | 0.005 | 1.59 (0.79-3.23) | 0.195 |
| ICM grade |  |  |  |  |
| A | reference |  | reference |  |
| B | 0.50 (0.37-0.75) | < 0.001 | 0.81 (0.51-1.29) | 0.383 |
| C | NA | NA | NA | NA |
| Trophectoderm grade |  |  |  |  |
| A | reference |  | reference |  |
| B | 0.53 (0.35-0.81) | 0.003 | 0.83 (0.50-1.37) | 0.459 |
| C | 0.06 (0.01-0.42) | 0.005 | 0.19 (0.02-1.55) | 0.122 |

Note: Grade ‘C’ in ICM morphology was excluded from the table because the number (N=11) was too small to obtain statistically meaningful results.
